# Forced-Air Convection Versus Underbody Conduction Warming Strategies to Maintain Perioperative Normothermia in Patients Undergoing Total Joint Arthroplasty

**DOI:** 10.7759/cureus.11474

**Published:** 2020-11-13

**Authors:** Robert McClain, Elird Bojaxhi, Samantha Ford, Karina Hex, Joseph Whalen, Christopher Robards

**Affiliations:** 1 Anesthesiology, Mayo Clinic, Jacksonville, USA; 2 Anesthesiology and Perioperative Medicine, Mayo Clinic, Jacksonville, USA; 3 Clinical Studies Unit, Mayo Clinic, Jacksonville, USA; 4 Orthopedics, Mayo Clinic, Jacksonville, USA

**Keywords:** arthroplasty, conductive warming, forced-air warming, perioperative hypothermia

## Abstract

Background

Forced-air warming is an established strategy for maintaining perioperative normothermia. However, this warming strategy can potentially contaminate the surgical field by circulating nonsterile air. This study aimed to determine whether changing practice away from this method resulted in non-inferior rates of perioperative hypothermia.

Methods

We performed a chart review of primary total hip and knee arthroplasty patients from 2014 to 2017, when the strategy of intraoperative forced-air warming (FAW) was changed to preoperative FAW along with intraoperative underbody conduction warming (CW) with an underbody warming mattress. Data included patient temperatures throughout all phases of care, blood loss and transfusion requirements, length of postanesthesia care unit (PACU) and hospital stays, and 30-day infection and mortality.

Results

A total of 769 charts were reviewed; 349 patients underwent surgery before the practice change and 420 after. Mean (SD; 95% CI) body temperatures at the time of incision were lower for group 1 than for group 2 (34.55 vs 35.52 °C [0.97 °C; 95% CI, 0.72-1.23 °C]). The average nadir of intraoperative body temperature was lower for group 1 than for group 2 (difference of means, 0.44 °C; 95% CI, 0.18-0.71 °C). Group 2 had a higher percentage of patients who presented hypothermic (temperature <36.0 °C) on arrival in the PACU (12.9% vs 7.7%).

Conclusion

Preoperative convective warming combined with intraoperative underbody conductive warming maintains normothermia during primary total joint arthroplasty and is non-inferior to forced-air intraoperative warming alone.

## Introduction

Inadvertent perioperative hypothermia is an important issue surrounding patients undergoing surgery. The numerous complications include increased intraoperative blood loss, increased postoperative wound infection rates, pressure ulcers, cardiac events, hospital costs, and increased lengths of stay [1-3]. Forced-air convection is an established method of perioperative temperature control for patients undergoing total joint replacement surgery [4-5]. However, reports have implicated the method in increased rates of surgical site infection (SSI) and periprosthetic joint infection (PPJI) [6-7]. Forced-air convection has the potential to circulate pathogenic microbes and to disrupt the downward linear airflow in the operating room, thus potentially resulting in increased infection rates [8-10]. Since SSI and PPJI are important contributors to patient morbidity and mortality, increased hospital lengths of stay, and health care expenditure, alternative warming strategies may need to be considered to maintain normothermia while avoiding the potential risk of infection by contamination of the surgical field [11-13].

At our institution, the standard practice of using intraoperative forced-air convection warming was changed to preoperative forced-air conduction and intraoperative underbody conduction warming on January 18, 2016. This quality improvement initiative and retrospective chart review sought to primarily determine whether this new practice resulted in the same or lower rates of hypothermia. A secondary outcome of interest was to compare the incidence of perioperative SSI and PPJI, intraoperative blood loss and blood transfusions, length of hospital and postanesthesia care unit (PACU) stay, and 30-day mortality between the two warming strategies.

## Materials and methods

The Mayo Clinic Institutional Review Board approved this retrospective chart review to assess the two perioperative warming strategies and the occurrence of hypothermia in patients undergoing hip or knee primary total joint arthroplasty at Mayo Clinic in Jacksonville, Florida, from January 1, 2014, to May 23, 2017. Before 2016, the warming strategy for these total joint replacements was intraoperative forced-air convection (group 1). In January 2016, our institution changed its practice to include preoperative forced-air convection and intraoperative underbody conduction warming (group 2). The change in practice was a multidisciplinary collaborative effort coordinated between the anesthesia practice, the orthopedic practice, and the perioperative nursing staff. All orthopedic surgeries were conducted in the same four dedicated orthopedic operating rooms by the same group of orthopedic surgeons and perioperative staff. The data from the month of January was omitted from the analysis since the practice was in transition. Data collected from the electronic health record included patients’ temperatures at the following times: in the preoperative holding area, at the time of the incision, when the intraoperative temperature was at its lowest, at the end of the surgery, and on arrival in the PACU. In addition, we recorded the length of PACU and hospital stays, the preoperative and postoperative hematocrits, the incidence and number of blood transfusions, the 1-year incidence of SSI and PPJI, and 30-day mortality. Only patients who underwent elective primary joint replacements were included.

Statistical analysis

This was a noninferiority study to compare the two warming techniques. Continuous variables were summarized as mean (SD), and categorical variables were reported as frequency (percentage) for the whole population and by groups. The differences in continuous outcome measurements were summarized as the difference of means (95% CI), and the differences in categorical outcome measurements were summarized as odds ratio (95% CI). Noninferiority was confirmed if the upper and lower sides of the 95% CI in favor of group 1 did not exceed the limit of the difference that investigators were willing to accept as “not inferior.” The upper limit of acceptable difference was set at 5%. Our choice to not use p-values to demonstrate statistical significance largely stems from our use of the non-inferiority study design. Had we used p-values, we would run into the following problem with addressing our hypothesis: It is theoretically possible to have a p-value <0.05 when temperatures in one group are either cooler or warmer than in the other, i.e., a statistically significant difference in temperatures between two groups would not indicate which group was better or worse. The Excel 2010 Analysis ToolPak add-in program (Microsoft Corporation, Redmond, Washington) was used for statistical analyses. If the recorded temperatures were not different between the two groups, the study would need 652 patients for 90% assurance that the upper limit of a 90% two-sided confidence interval would exclude a difference in favor of the standard group by more than 5% [14].

## Results

We reviewed the medical records and identified 769 sequential patients who underwent primary total joint replacement of either the hip or the knee from January 1, 2014, to May 23, 2017. Group 1 included 349 patients who were warmed with intraoperative forced-air convection, and group 2 included 420 patients who were warmed with preoperative forced air and intraoperative underbody conduction patient demographics were similar between the two warming strategies (Table 1).

**Table 1:** Patient demographics ASA, American Society of Anesthesiologists physical status classification

Characteristic	Group 1 (n=349)	Group 2 (n=420)	Total (N=769)
Age, y			
Mean (SD)	67.44 (12.07)	67.96 (10.89)	67.73 (11.44)
Range	15.00-91.00	23.00-95.00	15.00-95.00
Sex, No. (%)			
Female	203 (58.2)	249 (59.3)	452 (58.8)
Male	146 (41.8)	171 (40.7)	317 (41.2)
ASA score			
Mean (SD)	2.47 (0.52)	2.51 (0.55)	2.49 (0.54)
Range	1.00-4.00	1.00-4.00	1.00-4.00
Date of surgery			
Median	Feb 27, 2015	Apr 26, 2016	Feb 15, 2016
Range	Mar 21, 2014, through Dec 31, 2015	Feb 2, 2016, through Feb 7, 2017	Mar 21, 2014, through Feb 7, 2017

**Figure 1:**
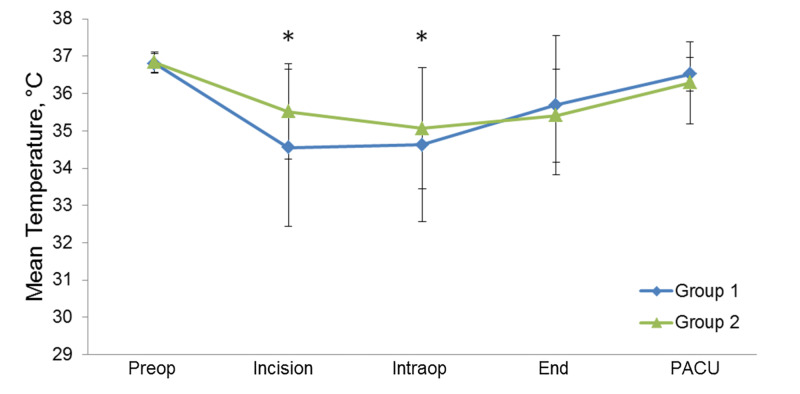
Mean body temperatures for the Patients Before (Group 1) and After (Group 2) the intervention The five time points were preoperatively (Preop), at the time of the incision (Incision), at the time of the lowest intraoperative temperature (Intraop), at the end of surgery (End), and on arrival in the postanesthesia care unit (PACU). Asterisks indicate statistical significance where the 95% CI does not include 0; whisker bars indicate SD.

**Figure 2:**
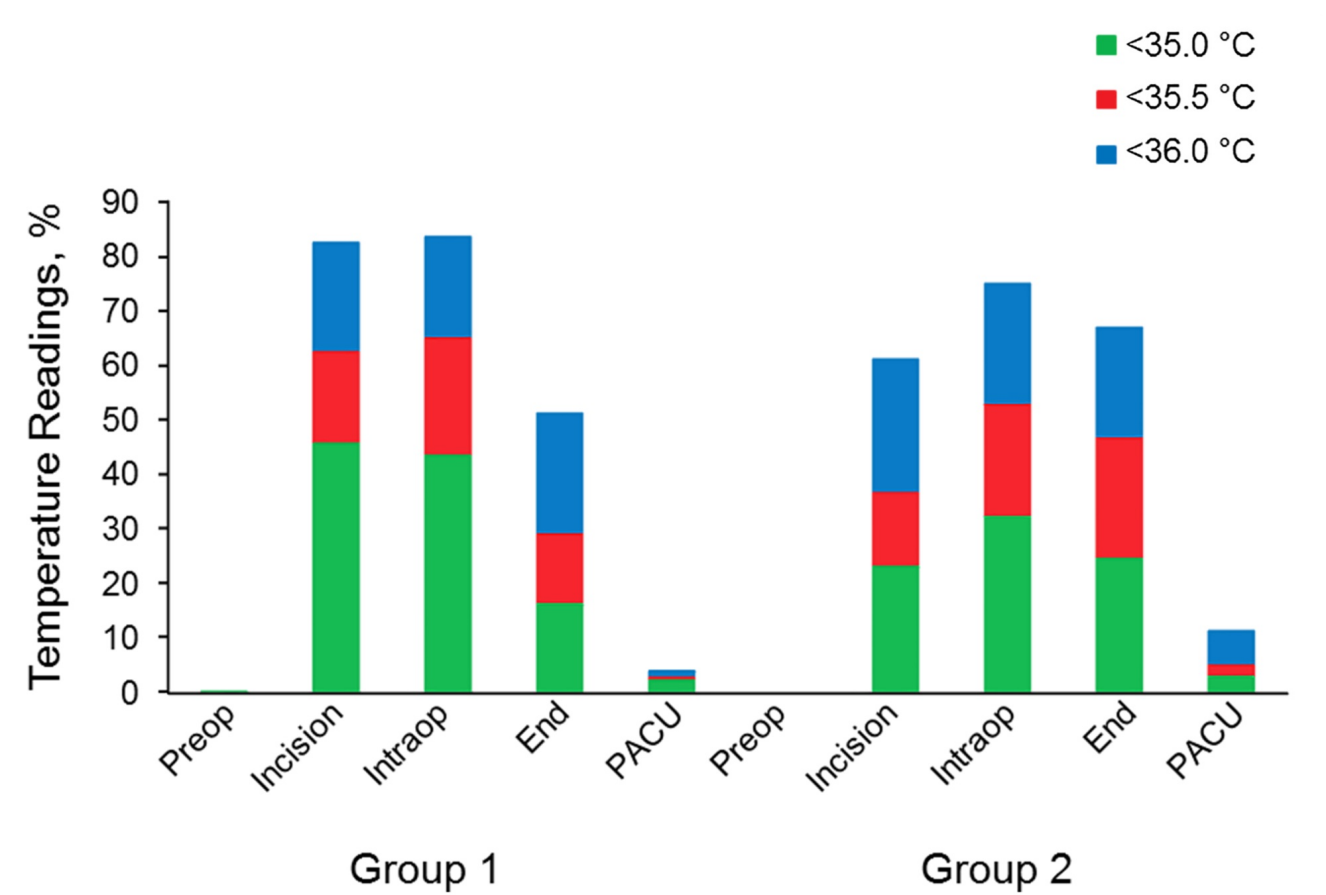
Percentage of temperature readings below the Threshold Before (Group 1) and After (Group 2) the intervention The five time points were preoperatively (Preop), at the time of the incision (Incision), at the time of the lowest intraoperative temperature (Intraop), at the end of surgery (End), and on arrival in the postanesthesia care unit (PACU).

Mean body temperature at the time of incision was lower for group 1 patients than for group 2 patients (34.55 °C vs 35.52 °C; difference of means, 0.97 °C; 95% CI, 0.72-1.23 °C) (considered statistically significant as the 95% CI does not include 0). The average nadir of intraoperative body temperature was 0.44 °C lower for group 1 than for group 2 (95% CI, 0.18-0.71) (Figure 1, Table 2). Group 2 had a higher percentage of patients who were hypothermic on arrival to PACU (12.9% vs 7.7%) (Figure 2).

**Table 2:** Temperatures Before (Group 1) and After (Group 2) the intervention PACU, postanesthesia care unit

Time of Temperature Reading	Group 1 Temperature, °C		Group 2 Temperature, °C		Difference of Means (Group 2 Minus Group 1) (95% CI), °C
	Mean	SD	Mean	SD	
Preoperative	36.81	0.27	36.84	0.27	0.03 (−0.01 to 0.07)
At incision	34.55	2.11	35.52	1.28	0.97 (0.72 to 1.23)
Intraoperative (lowest temperature)	34.63	2.06	35.07	1.63	0.44 (0.18 to 0.71)
At end of surgery	35.69	1.87	35.41	1.25	−0.28 (−0.44 to 0.01)
On arrival in PACU	36.52	0.45	36.29	1.10	−0.23 (−0.36 to 0.11)

Compared with patients in group 1, patients in group 2 stayed longer in the PACU (difference of means, 18.36 minutes; 95% CI, 10.45-26.27 minutes) (Table 3). Neither the infection data (SSI or PPJI) nor the mortality data showed any significant differences between the groups. Infection (SSI or PPJI) occurred in 10 patients (2.9%) in group 1 and in seven patients (1.7%) in group 2. The 30-day mortality was 1.1% (n=4) in group 1 and 0.5% (n=2) in group 2; among those who died, one patient (from group 1) had an infection (SSI or PPJI). Other comorbidities in the patients who died were older age (>80 years), obstructive biliary jaundice, ileus with abdominal compartment syndrome, malnutrition, diabetes mellitus, dementia, pulmonary hypertension, and end-stage renal disease. Length of stay in the hospital, preoperative and postoperative hematocrit values, and the number of blood transfusions were not significantly different between the two groups (Tables 3-4).

**Table 3:** Length of stay and hematocrit PACU, postanesthesia care unit

Parameter	Group 1, Mean (SD)	Group 2, Mean (SD)	Difference of Means (Group 2 Minus Group 1) (95% CI)
Length of stay			
PACU, min	99.44 (49.08)	117.80 (60.58)	18.36 (10.45 to 26.27)
Hospital, d	3.42 (3.91)	2.98 (1.55)	−0.44 (−0.85 to −0.03)
Hematocrit, vol%			
Preoperative	41.96 (16.59)	38.83 (17.74)	−3.13 (−5.58 to −0.68)
Postoperative	41.09 (37.12)	40.50 (35.75)	−0.59 (−5.78 to 4.61)

**Table 4:** Mortality, transfusions, hypothermia, and infections PACU, postanesthesia care unit; PPJI, periprosthetic joint infection; SSI, surgical site infection

Parameter	Patients, No. (%)		Odds Ratio (95% CI)
	Group 1	Group 2	
30-d mortality	4 (1.1)	2 (0.5)	0.41 (0.06-2.13)
Blood transfusion	7 (2.0)	8 (1.9)	0.95 (0.34-2.73)
Hypothermia			
On arrival in PACU	27 (7.7)	54 (12.9)	1.76 (1.09-2.9)
At end of surgery	58 (16.6)	107 (25.5)	1.72 (1.2-2.46)
Infection (SSI or PPJI)	10 (2.9)	7 (1.7)	0.57 (0.22-1.53)

## Discussion

Perioperative hypothermia is a well-established predictor of adverse events, and the prevention of hypothermia should be a focus of all aspects of perioperative patient care. Forced-air warming has been commonly used to maintain intraoperative normothermia, but concerns that it may affect operating room laminar airflow and lead to infections has led to an interest in other modalities. Currently, the literature is limited in evaluating CW, as an alternative strategy to FAW, and their respective impact of reducing surgical site infections potentially related to hypothermia. 

One study by Ralte et. al. [15] compared intraoperative forced-air warming with an underbody resistive warming mattress in a study of 90 patients undergoing shoulder arthroplasty randomly assigned between the two methods. The results showed that intraoperative forced-air warming maintained core body temperature better and that fewer patients in the forced-air group were hypothermic as compared with patients in the underbody resistive warming mattress group (13 of 47 patients vs 32 of 44 patients). In addition, core body temperatures increased after 30 minutes in the patients warmed with intraoperative forced air but not in the patients who were warmed with the underbody mattress. These results differ from ours, and this could be explained by the lack of preoperative active warming in the CW group and the different surgical population as reported by Ralte et al. Therefore, preoperative warming may play a major role in maintaining normothermia regardless of whether FAW or CW is used intraoperatively.

The effectiveness of preoperative warming has been well-described and our findings are consistent with those of previous studies [16-19]. In a case-control study of 60 patients undergoing hip or knee arthroplasty, Rosenkilde et al. showed that the incidence of unintended perioperative hypothermia was 13% for patients who received preoperative warming as compared with 43% for those who did not [17]. Our mixed results for hypothermia occurrences can be explained by understanding that a forced-air warming system is a more effective method to actively warm patients. The nadir temperature in group 1 patients was lower than in group 2 patients. This could be due to the absence of preoperative warming for group 1. Group 2 patients were warmer upon entry in the operating room and, in general, took longer to become hypothermic. However, group 1 patients (with a forced-air warmer) were warmed more effectively as demonstrated by their overall higher average temperatures on arrival in the PACU compared to group 2 patients (with the underbody warmer) but the difference was not statistically significant.

The shift away from intraoperative forced-air warming at our institution did not show a significant decrease in the rate of infection (SSI or PPJI) or mortality, although our study was not designed to detect such a difference. PACU length of stay was longer for group 2; this may be related to more patients in group 2 arriving in the PACU with hypothermia and thus requiring longer stays to meet discharge criteria (body temperature >36.0 °C). This may not be a relevant finding because the duration of PACU stay is multifactorial (depending on bed availability, pain control, postoperative nausea, and vomiting, etc.) and may not be directly related to temperature.

Limitations

From this study, we cannot directly compare intraoperative forced-air convection with underbody conduction because of the confounding variable of preoperative active warming in group 2. Another limitation is that we did not account for different temperature measurement methods (i.e., core vs skin and brand of temperature probe), and some amount of measurement variation may have occurred between the two groups. However, one can assume that variations in the measurement method would be evenly distributed in both groups because there was no change in practice with respect to temperature monitoring. Also, the type of anesthesia was not considered. Unless it is contraindicated or has failed, spinal anesthesia is used in our practice for patients undergoing hip or knee total joint arthroplasty. We did not distinguish between patients who received central neuraxial anesthesia and those who received general anesthesia. Thermoregulation with spinal anesthesia is physiologically different from thermoregulation with general endotracheal anesthesia, but it is unclear whether perioperative hypothermia is more likely to develop with either one. Thus, the differences in anesthesia in our study population may have skewed our results.

## Conclusions

The use of preoperative forced-air convection in combination with intraoperative conduction warming is non-inferior for the maintenance of normothermia as compared with the use of intraoperative forced-air convection alone. However, moving away from the use of intraoperative forced-air convection does not appear to decrease the risk of surgical infection.
